# Liraglutide improves peripheral perfusion and markers of angiogenesis and inflammation in people with type 2 diabetes and peripheral artery disease: An 18‐month follow‐up of a randomized clinical trial

**DOI:** 10.1111/dom.16419

**Published:** 2025-04-25

**Authors:** Paola Caruso, Maria Ida Maiorino, Miriam Longo, Antonietta Maio, Lorenzo Scappaticcio, Nicole Di Martino, Carla Carbone, Mariluce Barrasso, Mariangela Caputo, Maurizio Gicchino, Giuseppe Bellastella, Dario Giugliano, Katherine Esposito

**Affiliations:** ^1^ Division of Endocrinology and Metabolic Diseases University of Campania “Luigi Vanvitelli” Naples Italy; ^2^ Department of Advanced Medical and Surgical Sciences University of Campania “Luigi Vanvitelli” Naples Italy; ^3^ PhD Program of Translational Medicine, Department of Experimental Medicine University of Campania “Luigi Vanvitelli” Naples Italy

**Keywords:** angiogenesis markers circulating progenitor cells inflammatory markers Liraglutide, peripheral artery disease randomized clinical trial type 2 diabetes

## Abstract

**Aims:**

In a six‐month randomized clinical trial, improved peripheral perfusion has been shown with liraglutide, associated with favourable vascular effects in people with type 2 diabetes and peripheral artery disease (PAD). We aimed to evaluate the durability of these benefits and to elucidate some mechanisms underlying liraglutide's effect over an 18‐month follow‐up.

**Methods:**

STARDUST was a randomized clinical trial which compared liraglutide up to 1.8 mg/day with tailored therapeutic prescriptions to manage cardiovascular risk factors in 55 participants with type 2 diabetes and PAD. We report data of people who have reached the 18‐month follow‐up for the primary outcome (transcutaneous oxygen pressure, TcPO_2_) and also for additional secondary outcomes (markers of inflammation, angiogenesis and kidney function), as well as glycemic and metabolic parameters. TcPO_2_ was assessed with transcutaneous oximetry. Circulating levels of angiogenic progenitor cells and serum inflammation markers were evaluated by flow cytometry and enzyme‐linked immunosorbent assay, respectively.

**Results:**

Compared with the control group, significant differences favouring the liraglutide group were observed at 18 months for TcPO_2_ [estimated treated difference (95% CI), 10.9 mmHg (7.6 to 14.1 mmHg), *p* < 0.001]. At 18 months of follow‐up, participants in the liraglutide group, as compared with those in the control group, had a significant reduction in urine albumin to creatinine ratio (estimated difference, −103.9 mg/g Cr, 95%CI, −170.8 to −37.1, *p* = 0.003), C‐reactive protein (−0.5 mg/dL, 95%CI, −0.8 to −0.2, *p* = 0.002), as well as interleukin‐6 (−32.6 pg/mL, 95%CI, −54.6 to −10.5, *p* = 0.004). Compared with the control group, participants in the liraglutide group showed significantly higher concentrations of circulating progenitor cells and endothelial progenitor cells at both 6 and 18 months, for CD34^+^, CD133^+^, KDR^+^, CD34^+^/KDR^+^ and CD34^+^/CD133^+^/KDR^+^. Liraglutide was also associated with a higher increase in vascular endothelial growth factor A at 18 months (70.1 pg/mL, 95%CI, 44.7 to 95.4, *p* < 0.001).

**Conclusions:**

In people with type 2 diabetes and PAD, liraglutide increased peripheral perfusion, with amelioration of markers of angiogenesis and inflammation over an 18‐month follow‐up.

## 1 INTRODUCTION

More than 236 million people were affected by peripheral artery disease (PAD) worldwide in 2015.^1^ People with diabetes are nearly twice as likely to develop PAD than those without diabetes; moreover, PAD has been reported to be the most common initial manifestation of cardiovascular disease (CVD) in people with type 2 diabetes.^2^ Indeed, approximately 70% of cases undergoing lower extremity amputation in the United States are attributed to diabetes.^3^ Comorbid PAD is associated with significant increases in the risk of limb and CV adverse events, including major adverse cardiovascular events, heart failure outcomes, kidney disease complications, major adverse limb events (MALE), vascular hospitalizations and mortality.^4^ Current medications for PAD include vasodilators, antiplatelet and anticoagulant agents, glucose‐lowering medications to reduce the risk of CVD and renal events regardless of diabetes status and anti‐hypertensive and lipid‐lowering drugs.^5^, ^6^ The benefits of novel glucose‐lowering agents, such as glucagon‐like peptide‐1 receptor agonists (GLP‐1RA) and sodium‐glucose cotransporter‐2 inhibitors (SGLT‐2i), are not understood in full as existing data are often derived from one post hoc analysis of PAD subgroups or real‐world data.^7^, ^8^ GLP‐1RA show great promise for treating PAD in people with type 2 diabetes, since they may have systemic microcirculatory benefits in the peripheral vascular district, including reduced inflammation and oxidative stress, improved endothelial function, vasodilatation and anti‐atherosclerotic effects.^7^ Of note, a post hoc analysis of the LEADER trial reported that liraglutide was associated with a similar rate of peripheral revascularization and foot infections and a reduced risk of amputations as compared with placebo in individuals at high risk of cardiovascular events.^9^

In an open‐label randomized clinical trial of 55 patients with type 2 diabetes and PAD, we have shown that 1.8‐mg liraglutide daily, compared with management of main cardiovascular risk factors, improved peripheral perfusion suggesting that it may prevent the clinical progression of PAD.^10^ To further explore our understanding of the pleiotropic CV properties of liraglutide, we conducted a vascular biomarker analysis as a pre‐specified secondary outcome of the STARDUST trial. We report here the results of the extended follow‐up up to 18 months, in order to investigate the durability of the benefits and to elucidate some mechanisms underlying liraglutide's effect.

## 2 METHODS

STARDUST (Effects of the GLP‐1 receptor agonist Liraglutide on lower limb perfusion in people with type 2 diabetes and peripheral artery disease: An open‐label randomized clinical trial) is a single‐centre, open‐label randomized clinical trial registered on ClinicalTrials.gov (**NCT04881110**).

In brief, a total of 89 patients, 35 years or older with type 2 diabetes and a diagnosis of PAD were screened for eligibility between February 1, 2021, and June 30, 2022. Main inclusion criteria included a transcutaneous oxygen pressure (TcPO_2_) of the foot between 30 and 49 mmHg at the screening visit and HbA1c ranging from 6.5% to 8.0% (48 to 64 mmol/mol) while taking stable doses of glucose‐lowering medications, with the exclusion of current or previous therapy with GLP‐1RA, dipeptidyl peptidase 4 inhibitors or SGLT‐2i. The 60 individuals who met the inclusion criteria underwent randomization to liraglutide subcutaneous daily injection (*n* = 30) or a matching control group (*n* = 30).

Participants randomized to liraglutide received 0.6 mg once‐daily liraglutide subcutaneous injections at approximately the same time each day, with titration on a weekly schedule by a 0.6 mg increase to the target full dose of 1.8 mg. Individuals in both groups were given tailored therapeutic prescriptions of anti‐hypertensive and lipid‐lowering medications to manage cardiovascular risk factors: blood pressure (systolic <130 mmHg and diastolic <80 mmHg), low‐density lipoprotein cholesterol level below 70 mg/dL, aspirin (100 mg/day) or clopidogrel (75 mg/day).

Three individuals in the liraglutide group and 2 in the control group withdrew from the study and/or were lost during follow‐up: 55 participants completed the follow‐up until December 31, 2022, and were included in the analysis at 6 months. Participants in the study, according to the protocol, continued to follow therapeutic prescriptions and underwent regular follow‐up visits over the 18‐month period. The medical staff involved in the intervention enrolled participants, assigned them to the trial groups and conducted the follow‐up visits at the hospital, whereas the laboratory staff did not know the patients' group assignments. The trial staff who assessed outcomes and analysed the data were also masked to group assignment.

### 2.1 Outcomes

Change in TcPO_2_ at 6 months was one of the primary outcomes of the trial. Here, we report data of people who have reached the 18‐month follow‐up for the coprimary outcome and also for additional secondary outcomes, including markers of inflammation (C‐reactive protein [CRP], tumour necrosis factor α [TNF‐α], interleukin‐6 [IL‐6]), markers of angiogenesis (circulating progenitor cells [CPCs, CD34^+^, CD133^+^, CD34^+^/CD133^+^] and endothelial progenitor cells [EPCs, KDR^+^, CD34^+^/KDR^+^, CD133^+^/KDR^+^, CD34^+^/CD133^+^/KDR^+^], vascular endothelial growth factor A [VEGF‐A]), markers of kidney function (urine albumin to creatinine ratio [UACR] and estimated glomerular filtration rate [eGFR]), as well as glycaemic and metabolic parameters (HbA1c, weight and systolic and diastolic blood pressure), lipid profile (total cholesterol, high‐density lipoprotein and low‐density lipoprotein cholesterol and triglycerides) and ankle‐brachial index (ABI). The study complies with the Declaration of Helsinki and the International Conference on Harmonization/Good Clinical Practice Guidelines. All participants signed an informed consent.

### 2.2 Evaluation of PAD

TcPO_2_ was assessed with transcutaneous oximetry (Perimed Inc) at the districts of medial malleolus and the dorsum of the foot, irrigated by posterior and anterior tibial arteries, respectively. After a 20‐min supine rest, the electrochemical transducer was fixed on the skin, avoiding areas overlying bone or superficial vessels. The device was calibrated before each use for at least 5 minutes. The assessment lasted 16 minutes and was performed in both limbs. The lowest value was used for the analysis. Ankle‐brachial index evaluation was obtained by dividing the leg systolic pressure by the arm systolic pressure, both detected through a Doppler probe and a sphygmomanometer.

### 2.3 CPCs and EPCs

Assessment of levels of CPCs and EPCs was performed on fresh blood samples collected in citrate tubes after overnight fasting. Peripheral blood cells were analysed for the expression of surface antigens CD34, KDR and CD133 by direct flow cytometry.^11^ CD34^+^/CD133^+^ cells were defined as cells presenting the two antigens in the mononuclear cell population. Then, we examined the cell population for the dual expression of KDR. Cells presenting all three antigens were identified by the simultaneous expression of KDR and CD133 in the CD34^+^ gate. We defined CPCs the cellular pool expressing the markers of stem cells (CD34^+^, CD133^+^ and CD34^+^/CD133^+^ cells), and EPCs as all cells presenting KDR that suggest their commitment toward the endothelial line (CD34^+^/KDR^+^, CD133^+^/KDR^+^ and CD34^+^/CD133^+^/KDR^+^ cells). Data were processed with the use of the Macintosh CELL Quest software programme (Becton‐Dickinson). Both CPCs and EPCs counts were expressed as the number of cells per 10^6^.

### 2.4 Other parameters

CRP (high sensitivity), TNF‐α, IL‐6 and VEGF‐A (Quantikine; R&D Systems, Minneapolis, USA) were assessed by enzyme‐linked immunosorbent assay (ELISA). Samples were assayed in duplicate and re‐run if duplicates differed by >20%. The other parameters were measured according to the central laboratory.

### 2.5 Statistical analysis

The sample size calculation has been reported in the study protocol.

Descriptive statistics were used for demographic and baseline clinical characteristics of all participants in the study. Continuous variables were described as either means (SDs) or medians (IQRs) according to sample distribution, and count data were reported as numbers (percentages). Comparisons of baseline data between groups were performed by Student's t test or Mann–Whitney Rank Sum test, depending on the normality of sample distribution. Comparison among frequencies was made with the χ^2^ test.

The paired t test and Wilcoxon signed rank test within each patients' group were used to assess changes in the studied variables from baseline to 6 or 18 months. A 2‐sample t test was used to compare differences between the interventions. Data were also analysed by analysis of variance for repeated measures with a post hoc comparisons made with the Scheffé's test. A 2‐sided *p* < 0.05 was considered statistically significant. All analyses were conducted using SPSS software, version 24.0 (SPSS Inc).

## 3 RESULTS

Baseline characteristics of the two groups are reported in Table 1. The diagnosis of PAD was done through Doppler ultrasonography in 10 participants of the Liraglutide group and in 11 participants of the control group, through computed tomography angiography in 8 and 7 participants and through angiography in 9 and 10 participants, respectively (ESM Table 1). More than threequarters of participants were male (78%) and their glycaemic control was fair as judged by their mean level of HbA1c.

**TABLE 1:** Baseline characteristics.

	Patients (55)	
Parameters	Liraglutide (27)	Control (28)
Age, years	67.2 ± 8.3	67.4 ± 8.9
Diabetes duration, years	15.2 ± 11.8	16.5 ± 13.9
HbA_1c_, %	7.1 ± 0.5	6.8 ± 0.8
HbA_1c_, mmol/mol	54 ± 6	51 ± 9
Weight, kg	80.9 ± 14.3	81.9 ± 13.5
Blood Pressure, mmHg		
SBP	130.0 (130.0, 140.0)	140.0 (130.0, 140.0)
DBP	70.0 (70.0, 80.0)	80.0 (75.0, 80.0)
Renal function		
eGFR, mL/min	73.4 ± 20.6	74.1 ± 15.5
UACR, mg/g Cr	47.4 (24.1, 117.5)	60.0 (39.0, 74.3)
Lipids, mg/dL		
Total cholesterol	143.5 ± 48.6	155.2 ± 35.3
HDL‐cholesterol	45.9 ± 14.5	45.0 ± 9.7
LDL‐cholesterol	76.7 ± 37.6	85.6 ± 28.1
Triglycerides	104.5 (80.0, 133.0)	108.0 (80.0, 145.0)
TcPO_2_, mmHg	40.0 ± 5.9	40.5 ± 5.7
ABI	0.9 (0.9, 1.1)	0.9 (0.9, 1.1)
Inflammation markers		
CRP, mg/dL	0.4 (0.2, 1.5)	0.4 (0.1, 1.8)
IL‐6, pg/mL	44.7 (36.9, 63.9)	43.9 (29.4, 60.5)
TNF‐α, pg/mL	9.1 (4.1, 88.5)	9.6 (3.8, 79.1)
Angiogenesis markers		
CD34+, *n* × 10^6^	148.0 (120.5, 191.0)	178.0 (131.0, 221.0)
CD133+, *n* × 10^6^	191.0 (140.0, 255.5)	241.0 (188.0, 299.0)
KDR+, *n* × 10^6^	100.0 (53.0, 124.0)	92.0 (65.0, 110.0)
CD34+/CD133+, *n* × 10^6^	89.5 (51.5, 122.5)	101.0 (82.00, 124.0)
CD34+/KDR+, *n* × 10^6^	8.0 (6.5, 22.5)	12.0 (7.0, 17. 0)
CD133+/KDR+, *n* × 10^6^	5.5 (3.0, 9.5)	3.0 (3.0, 6.0)
CD34+/CD133+/KDR+, *n* × 10^6^	4.0 (1.5, 5.0)	2.0 (1.0, 4.0)
VEGF‐A, pg/mL	73.1 (47.2, 141.0)	71.0 (59.2, 116.5)

*Note*: Data are presented as mean ± SD or median (IQR).

Abbreviations: ABI, ankle‐brachial index; CRP, C‐reactive protein; DBP, diastolic blood pressure; eGFR, estimated glomerular filtration rate; HbA1c, glycated haemoglobin; IL‐6, interleukin 6; SBP, systolic blood pressure; TcPO2, transcutaneous oxygen pressure; TNF‐α, tumour necrosis factor α; UACR, urinary albumin to creatinine ratio; VEGF‐A, vascular endothelial growth factor A.

Significant differences from baseline were recorded mostly within the liraglutide group (Table 2).

**TABLE 2:** Primary and secondary outcomes after 6 and 18 months.

	Patients (55)			
	6 months		18 months	
Parameters	Liraglutide (27)	Control (28)	Liraglutide (27)	Control (28)
TcPO_2_, mmHg	54.2 ± 5.9^§^	43.4 ± 4.7^§^	54.2 ± 5.7^§^	43.8 ± 4.5^§^
ABI	0.9 ± 0.1	0.9 ± 0.1	0.9 ± 0.1	1.0 ± 0.1
HbA_1c_, %	6.7 ± 0.7*	6.8 ± 0.8	6.6 ± 0.5^+^	6.8 ± 0.7
HbA_1c_, mmol/mol	50 ± 8*	51 ± 9	49 ± 6^+^	51 ± 8
Weight, kg	78.5 ± 14.4*	80.7 ± 12.5	78.1 ± 13.9^§^	80.5 ± 12.8
Blood Pressure, mmHg				
SBP	130.0 (120.0, 130.0)^+^	130.0 (125.0, 140.0)^+^	127.5 (125.0, 130.0)^+^	130.0 (130.0, 130.0)^+^
DBP	75.0 (70.0, 80.0)	80.0 (70.0, 80.0)	80.0 (70.0, 80.0)	80.0 (70.0, 80.0)
Renal function				
eGFR, mL/min	71.0 ± 18.4	74.7 ± 13.4	73.4 ± 18.5	72.4 ± 12.6
UACR, mg/g Cr	16.0 (14.5, 27.1)^+^	54.0 (17.0, 131.0)	16.9 (9.5, 23.3)^+^	48.7 (22.0, 120.0)
Lipids, mg/dL				
Total cholesterol	142.3 ± 40.7	158.8 ± 55.2	133.3 ± 36.6	138.7 ± 32.6^+^
HDL‐cholesterol	47.1 ± 17.6	45.1 ± 9.6	49.9 ± 15.2^+^	46.6 ± 7.6
LDL‐cholesterol	71.9 ± 30.9	94.4 ± 49.3	64.9 ± 17.8	72.8 ± 14.1^+^
Triglycerides	113.0 (89.5, 142.5)	109.0 (91.0, 126.0)	102.0 (85.0, 126.5)	108.5 (91.0, 130.0)^+^
Inflammation markers				
CRP, mg/dL	0.3 (0.1, 0.5)^§^	0.9 (0.2, 1.2)	0.3 (0.1, 0.7)^+^	0.7 (0.3, 1.5)
IL‐6, pg/mL	4.0 (0.1, 10.3)^§^	29.8 (6.0, 48.0)^§^	7.2 (3.6, 12.5)^§^	28.6 (9.2, 42.2)^§^
TNF‐α, pg/mL	1.9 (0.1, 9.8)^§^	6.5 (2.4, 12.4)^+^	2.9 (0.4, 6.5)^§^	6.1 (2.1, 11.1)^+^
Angiogenesis markers				
CD34+, *n* × 10^6^	250.0 (163.5, 346.0)^§^	223.5 (133.0, 258.0)	235.0 (169.5, 345.0)^§^	190.5 (134.0, 247.0)
CD133+, *n* × 10^6^	280.5 (239.0, 367.0)^§^	225.0 (165.0, 386.0)	257.5 (217.0, 343.5)^§^	242.5 (183.0, 375.0)
KDR+, *n* × 10^6^	147.5 (115.0, 198.5)^+^	74.0 (53.0, 132.0)	140.5 (123.0, 161.5)^§^	78.0 (69.0, 120.0)
CD34+/CD133+, *n* × 10^6^	93.5 (73.0, 128.0)	104.0 (60.0, 153.0)	110.0 (73.5, 141.5)^+^	129.0 (95.0, 148.0)
CD34+/KDR+, *n* × 10^6^	21.0 (14.5, 52.0)^§^	18.0 (10.0, 26.0)^+^	19.5 (14.0, 46.5)^§^	18.5 (10.0, 24.0)^+^
CD133+/KDR+, *n* × 10^6^	11.0 (5.5, 14.0)^§^	6.0 (4.0, 11.0)^§^	8.5 (6.5, 12.5)^+^	5.0 (4.0, 10.0)^§^
CD34+/CD133+/KDR+, *n* × 10^6^	7.5 (6.0, 10.0)^§^	6.0 (2.0, 6.0)^+^	7.0 (5.5, 11.0)^§^	4.5 (2.0, 6.0)^§^
VEGF‐A, pg/mL	124.0 (77.5, 188.0)^§^	68.4 (52.2, 80.8)^+^	120.5 (78.2, 182.0)^§^	76.5 (60.4, 104.3)

*Note*: Data are presented as mean ± SD or median (IQR). Significant differences versus baseline (Table 1) within groups: * = *p* < 0.5; ^+^ = *p* ≤ 0.1; ^§^ = *p* < 0.001.

Abbreviations: ABI, ankle‐brachial index; CRP, C‐reactive protein; DBP, diastolic blood pressure; eGFR, estimated glomerular filtration rate; HbA1c, glycated haemoglobin; IL‐6, interleukin 6; SBP, systolic blood pressure; TcPO_2_, transcutaneous oxygen pressure; TNF‐α, tumour necrosis factor α; UACR, urinary albumin to creatinine ratio; VEGF‐A, vascular endothelial growth factor A.

TcPO_2_ increased in both groups at 6 months and then stabilized thereafter (Table 2). Significant differences favouring the liraglutide group, as compared with the control group, were observed at 6 months (Table 3) and at 18 months (Table 4). Except for UACR, which showed a significant decrease at 6 and 18 months (*p* = 0.003 for both) in the liraglutide group as compared with the control group (Tables 3 and 4), no other significant differences between groups were observed in HbA1c, body weight, blood pressure, lipid levels, eGFR and ABI.

**TABLE 3:** Comparison of the changes in primary and additional outcomes between groups after 6 months.

	Liraglutide	Control	Estimated treatment difference (95% CI)	*p* value
*Primary outcome*				
TcPO_2_, mmHg	14.2 (7.1)	2.9 (3.8)	11.2 (8.0 to 14.5)	<0.001
*Secondary outcome*				
Renal function				
UACR, mg/g Cr	−90.5 (161.9)	28.8 (98.5)	−119.4 (−195.0 to −43.8)	0.003
Inflammation markers				
CRP, mg/dL	−0.4 (0.5)	−0.03 (0.6)	−0.4 (−0.7 to −0.07)	0.018
IL‐6, pg/mL	−48.0 (55.5)	−16.3 (23.0)	−31.6 (−54.6 to −8.7)	0.008
TNF‐α, pg/mL	−31.7 (40.5)	−27.2 (40.6)	−4.4 (−26.6 to 17.6)	0.686
Angiogenesis markers				
CD34+, *n* × 10^6^	99.8 (119.4)	33.5 (99.1)	66.3 (4.1 to 128.5)	0.037
CD133+, *n* × 10^6^	106.2 (111.4)	29.6 (151.1)	76.6 (0.6 to 152.6)	0.048
KDR+, *n* × 10^6^	54.7 (73.7)	8.6 (84.9)	46.1 (0.7 to 91.4)	0.047
CD34+/CD133+, *n* × 10^6^	11.9 (56.3)	5.4 (73.1)	6.4 (30.8 to 43.8)	0.728
CD34+/KDR+, *n* × 10^6^	14.8 (14.8)	6.5 (11.6)	8.3 (0.7 to 15.8)	0.032
CD133+/KDR+, *n* × 10^6^	4.4 (5.7)	2.7 (3.0)	1.6 (0.9 to 4.2)	0.206
CD34+/CD133+/KDR+, *n* × 10^6^	4.1 (3.4)	2.0 (2.7)	2.1 (0.4 to 3.9)	0.017
VEGF‐A, pg/mL	71.3 (67.4)	−16.2 (26.2)	87.5 (59.9 to 115.1)	<0.001

*Note*: Δ refers to the difference between values measured after 6 months versus baseline within each group. Outcome values are expressed as mean (SD).

Abbreviations: CRP, C‐reactive protein; IL‐6, interleukin 6; TcPO_2_, transcutaneous oxygen pressure; TNF‐α, tumour necrosis factor α; UACR, urinary albumin to creatinine ratio; VEGF‐A, vascular endothelial growth factor A.

**TABLE 4:** Comparison of the changes of primary and additional outcomes between groups after 18 months.

	Liraglutide	Control	Estimated treatment difference (95% CI)	*p* value
*Primary outcome*				
TcPO_2_, mmHg	14.2 (7.2)	3.3 (3.7)	10.9 (7.6 to 14.1)	<0.001
*Secondary outcome*				
Renal function				
UACR, mg/g Cr	−95.5 (159.5)	8.3 (55.6)	−103.9 (−170.8 to −37.1)	0.003
Inflammation markers				
CRP, mg/dL	−0.4 (0.6)	−0.05 (0.5)	−0.5 (−0.8 to −0.2)	0.002
IL‐6, pg/mL	−49.3 (54.6)	−16.8 (19.0)	−32.6 (−54.6 to −10.5)	0.004
TNF‐α, pg/mL	−32.9 (43.3)	−28.9 (42.3)	−3.9 (−27.4 to 19.4)	0.734
Angiogenesis markers				
CD34+, *n* × 10^6^	94.3 (112.0)	17.3 (78.8)	77.1 (22.3 to 131.8)	0.007
CD133+, *n* × 10^6^	72.5 (82.0)	21.4 (93.2)	51.0 (0.9 to 101.1)	0.046
KDR+, *n* × 10^6^	45.4 (54.5)	15.7 (61.8)	35.8 (2.5 to 69.0)	0.036
CD34+/CD13+, *n* × 10^6^3	16.8 (36.6)	15.7 (43.6)	1.1 (23.2 to 25.5)	0.925
CD34+/KDR+, *n* × 10^6^	12.7 (12.3)	6.0 (8.6)	6.7 (0.7 to 12.7)	0.029
CD133+/KDR+, *n* × 10^6^	2.8 (4.0)	2.4 (2.8)	0.4 (1.5 to 2.4)	0.676
CD34+/CD133+/KDR+, *n* × 10^6^	3.7 (2.8)	1.8 (2.0)	1.9 (0.5 to 3.3)	0.009
VEGF‐A, pg/mL	65.6 (63.9)	−4.5 (18.7)	70.1 (44.7 to 95.4)	<0.001

*Note*: Δ refers to the difference between values measured after 18 months versus baseline within each group. Outcome values are expressed as mean (SD).

Abbreviations: CRP, C‐reactive protein; IL‐6, interleukin 6; TcPO_2_, transcutaneous oxygen pressure; TNF‐α, tumour necrosis factor α; UACR, urinary albumin to creatinine ratio; VEGF‐A, vascular endothelial growth factor A.

Compared with the control group, participants randomized to the liraglutide group had a significant reduction of CRP at 6 (estimated treatment difference, −0.4 mg/dL, 95%CI, −0.7 to −0.007 mg/dL, *p* = 0.018) and 18 months (−0.5 mg/dL, −0.8 to −0.2 mg/dL, *p* = 0.002), as well as of IL‐6 (difference at 6 months, −31.6 pg/mL, 95%CI, −54.6 to −8.7 pg/mL, *p* = 0.008; difference at 18 months, −32.6 pg/mL, −54.6 to −10.5 pg/m, *p* = 0.004) (Tables 3 and 4). No significant differences between groups were found for TNF‐α concentrations.

**FIGURE 1:**
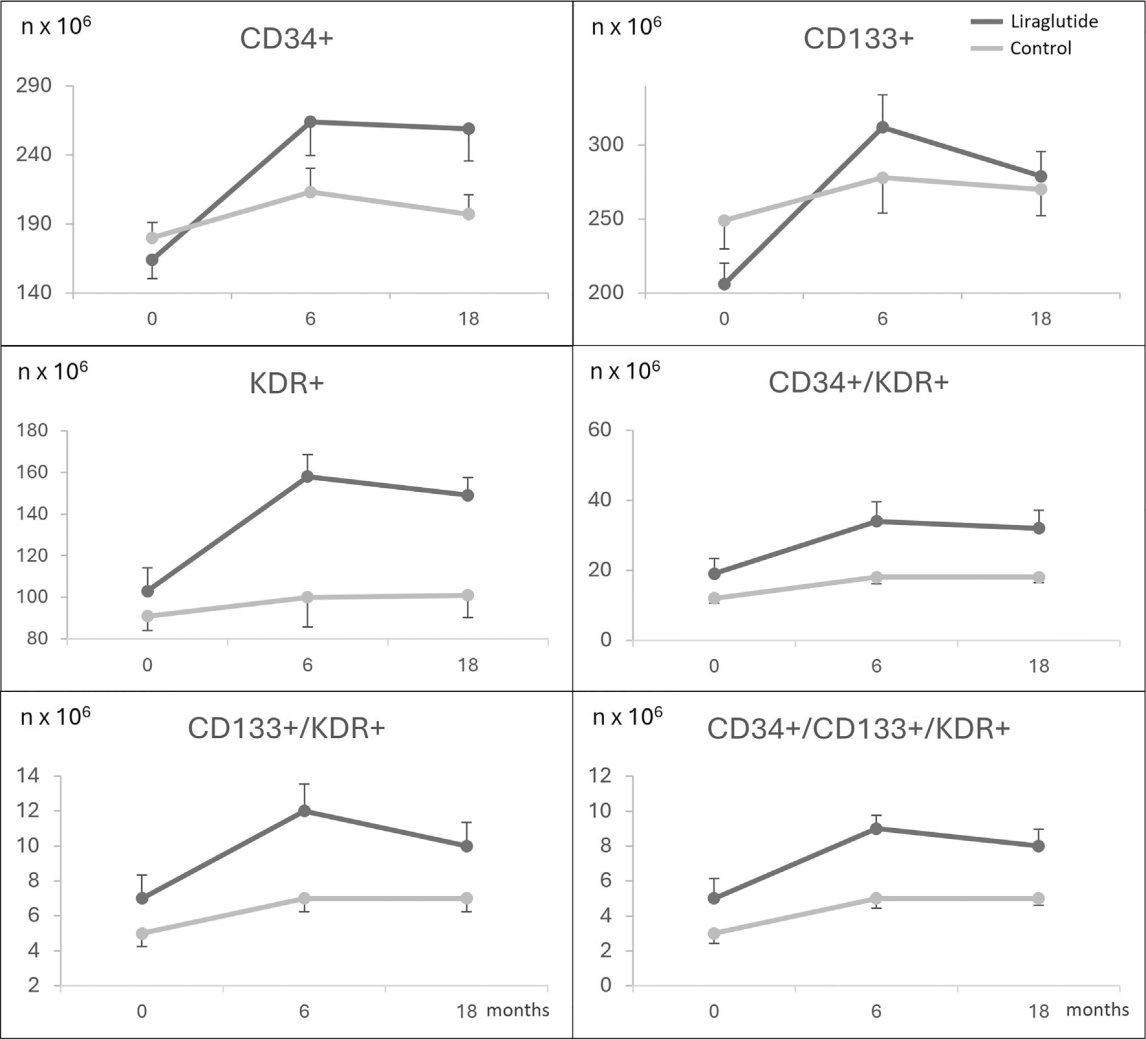
Mean change in CPCs and EPCs over time in the liraglutide group and control group. For each timepoint, error bars refer to the standard error.

Compared with the control group, participants of the liraglutide group showed significantly higher concentrations of CPCs and EPCs at both 6 and 18 months, for CD34^+^, CD133^+^, KDR^+^, CD34^+^/KDR^+^ and CD34^+^/CD133^+^/KDR^+^ (Figure 1, Tables 3 and 4). Liraglutide was also associated with a higher increase in VEGF‐A (difference at 6 months, 87.5 pg/mL, 95%CI, 59.9 to 115.1 pg/mL, *p* < 0.001; difference at 18 months, 70.1 pg/mL, 44.7 to 95.4 pg/mL, *p* < 0.001) (Tables 3 and 4).

No participants in either group reported adverse events to any medication administered.

## 4 DISCUSSION

The extended follow‐up of STARDUST at 18 months confirms the increase of peripheral perfusion in people with type 2 diabetes and PAD treated with 1.8‐mg liraglutide as compared with people who managed their CV risk factors. Moreover, the analysis of biomarkers of inflammation and angiogenesis shows benefits with liraglutide, including a reduction of CRP and IL‐6, associated with an increase in VEGF‐A and some CPCs or EPCs: in particular, circulating levels of CD34^+^, CD133^+^, KDR^+^, CD34^+^/KDR^+^ and CD34^+^/CD133^+^/KDR+ were significantly higher in the liraglutide group as compared with the control group. Taken together with the marked reduction of albuminuria, all this indicates a sustained effect of liraglutide in improving peripheral perfusion associated with multiple benefits on vascular health.

This study has many strengths, including the 18‐month duration of follow‐up, the assessment of peripheral perfusion through TcPO_2_, the naïve nature of patients to GLP‐1RA and the fair cardiometabolic control of risk factors. The study has also limitations which include its open‐label nature, partly mitigated by the blinding of clinical and laboratory outcomes and the relatively small size due to the strict entry criteria. Moreover, although consistent with PAD epidemiology, the number of women in the study is small; this could have introduced a potential sex‐related bias, which may reduce the external validity of the results.

Previous clinical trials have shown that liraglutide reduced albuminuria,^12^ which is thought to be a biomarker of microvascular and macrovascular endothelial dysfunction,^13^ as well as circulating levels of various inflammatory markers, including CRP, TNF‐α and IL‐6 in individuals with type 2 diabetes.^14^, ^15^, ^16^ Our results confirm these effects of liraglutide in people with type 2 diabetes and PAD, and also extend the previous observations demonstrating that they last for the entire 18‐month period of liraglutide administration.

The results of human studies assessing the role of liraglutide on circulating markers of angiogenesis are scanty. An increase in VEGF following liraglutide therapy has been suggested to be mediated via the CNPY2‐PERK pathway, involved with endothelial cell angiogenesis following hypoxia‐induced injury in animal models.^17^ VEGF is also implicated in endothelial cell expansion, proliferation and migration related to angiogenesis^18^ and thus may mitigate the effects of large vessel atherosclerosis. Moreover, in a randomized trial in 61 adults with type 2 diabetes, VEGF levels were higher at 26 weeks with liraglutide as compared with sitagliptin.^19^

Even more fragmented are the results of liraglutide on CPCs and EPCs. In a randomized, open‐label, active comparator trial assessing the effects of 26 weeks of liraglutide or sitagliptin on cardiovascular function in young adults with obesity and recently diagnosed type 2 diabetes, no significant change from baseline in CPCs with liraglutide was found.^19^ A previous small study in seven individuals with type 2 diabetes, exploring the short term (4 weeks) impact of liraglutide, reported no increase in EPCs.^20^ It is difficult to draw firm conclusions from these studies due to the variability in study design, duration and methodology used. However, the patients enrolled in STARDUST were older, with a long duration of disease and affected by PAD. Moreover, people with diabetes have been reported to have a reduction in the levels of circulating CPCs, which is strongly associated with future risk for multi‐organ damage, including micro‐ and macro‐angiopathy.^21^

CPCs are generally defined based on the surface expression of the haematopoietic stem cell markers CD34 and CD133. EPCs are phenotypes with vascular endothelial specification, account for ≤15% of CPCs and are characterized by the coexpression of endothelial markers (mostly the type 2 vascular endothelial growth factor receptor KDR). When phenotype‐specific risk estimates were pooled together, the overall risk ratio indicated that a low CPCs/EPCs count was associated with a significant 97% higher risk of future CV events.^22^ In addition, low circulating levels of CD34+/KDR+ EPCs predicted the occurrence of cardiovascular events and death from cardiovascular causes in patients at high cardiovascular risk.^23^

Among the CPCs, CD34^+^/CD133^+^ cells did not raise in the liraglutide group. A potential reason for this finding includes their haematopoietic origin, as indicated by the expression of CD133, which is selectively expressed on haematopoietic stem and progenitor cells. Of note, the unchanged levels of this cellular pool may reflect the rate of extra‐medullary haematopoiesis in the spleen, which could provide another peripheral reservoir of haematopoietic stem cells.^22^

The mounting evidence available in the literature indicates the close relationships between reduced levels of CPCs and adverse cardiovascular outcomes in different cohorts of patients. However, this may occur over a long period of time. In the Joslin Medalist study,^24^ the highest EPC levels were found in Medalists (patients with duration of diabetes of 50 years or longer) without cardiovascular diseases or diabetic microvascular complications, and this was consistent with the unusual survival of these individuals. Moreover, a longitudinal study of 187 patients with type 2 diabetes reported that a reduced baseline level of CD34^+^ cells predicted microvascular outcomes after a mean follow‐up of 3.9 years.^25^

Recent guidelines recommend the use of GLP‐1RA and/or SGLT‐2i to reduce CV risk.^5^ Concerns have been raised regarding the safety of SGLT‐2i in terms of limb events due to the increased incidence of amputations observed in the CANVAS (Canagliflozin CardioVascular Assessment Study) trial.^26^ Although meta‐analyses of cardiovascular outcomes trials reported neutral effects of SGLT‐2i on amputations,^27^, ^28^ real‐world data continue to appear showing an increased risk of major adverse limb effects (MALE) with SGLT‐2i as compared with GLP‐1RA.^29^, ^30^, ^31^ Whether these differences are due to a detrimental effect of SGLT‐2i, a beneficial effect of GLP‐1RA or both is unclear. It is possible that SGLT‐2i do not increase the risk of lower extremity amputation but rather that GLP‐1RA reduce this risk, which is much higher in patients with PAD.^32^ However, a post hoc analysis of the Liraglutide Effect and Action in Diabetes: Evaluation of Cardiovascular Outcome Results (LEADER) trial^9^ found that liraglutide was associated with reduced amputations compared with placebo. Moreover, in a national large cohort of high‐risk veterans with type 2 diabetes, SGLT‐2i use, as compared with dipeptidyl peptidase‐4 inhibitors use, was associated with an 18% increase in cause‐specific hazard of a composite PAD surgical outcome.^33^

People with diabetes and PAD have a fivefold increased risk of amputation and a threefold increased risk of death relative to their non‐diabetic counterparts.^34^ Real‐world data^28^, ^29^, ^30^ and the STARDUST results^10^ support the use of GLP‐1RA, in particular liraglutide, to prevent the clinical progression of PAD. The results from the ongoing STRIDE trial will tell us whether semaglutide may also improve functional capacity,^35^ although this has already been reported with liraglutide that is associated with a 25 m increase of 6‐minute walking distance at 6 months.^10^

In conclusion, the extended follow‐up of STARDUST shows that liraglutide increased peripheral perfusion in people with type 2 diabetes and PAD, associated with amelioration of markers of angiogenesis and inflammation. Since our findings do not allow us to derive a direct causality between the aforementioned mechanistic effects and limb outcomes, this relationship should be investigated in further studies, including also a larger number of women. GLP‐1RA These effects are persistent with continuous use of liraglutide, as GLP‐1 RAs are intended for long‐term management of the chronic condition of type 2 diabetes. Unfortunately, real‐world estimates for GLP‐1 RA discontinuation are in the range of 50% to 75% at 12 months.^36^

## AUTHOR CONTRIBUTIONS

PC, MIM and DG formulated the study question and design, performed the statistical analyses, interpreted the results and wrote the complete first draft of the manuscript. ML, LS, CC, MB, MC, MG, GB and KE revised and edited the manuscript and made substantial contributions to the interpretation of data. AM and NDM assisted in statistical analyses and performed laboratory analyses. All authors participated in the critical revision of the manuscript and approved the final version of the manuscript. The corresponding author (MIM) and senior author (KE) take full responsibility for the work and/or the conduct of the study, had access to the data and controlled the decision to publish.

## FUNDING INFORMATION

The trial was partially funded by the PhD program of Translational Medicine at the University of Campania “Luigi Vanvitelli”, Naples, Italy (Dr. Caruso). The funding source had no role in the design and conduct of the study; collection, management, analysis and interpretation of the data; preparation, review or approval of the manuscript; and the decision to submit the manuscript for publication.

## CONFLICT OF INTEREST STATEMENT

DG received a consultancy fee from Eli Lilly and has given lectures from Eli Lilly, Sanofi, Novartis, Astrazeneca and Novo Nordisk. MIM has given lectures for Novo Nordisk, Eli Lilly, and Sanofi. KE received a consultancy fee from Eli Lilly and has given lectures from Eli Lilly, Sanofi, Novo Nordisk, Roche, Bayer and Lifescan. All other authors declare that there are no relationships or activities that might bias, or be perceived to bias, their work.

## PEER REVIEW

The peer review history for this article is available at https://www.webofscience.com/api/gateway/wos/peer-review/10.1111/dom.16419.

## Supporting information


**Data S1.** Supporting Information.

## Data Availability

Mean data will be shared with bona fide researchers who submit a research proposal approved by the independent review board. Data will be made available after research completion.
