# Supplementary material for: Liraglutide improves peripheral perfusion and markers of angiogenesis and inflammation in people with type 2 diabetes and peripheral artery disease: An 18‐month follow‐up of a randomized clinical trial
Source: Diabetes Obes Metab. 2025 Apr 25;27(7):3891–900. doi: 10.1111/dom.16419 (PMC12146450; doi:10.1111/dom.16419)
Supplement: Supplementary file 1 — Data S1. Supporting Information. [file DOM-27-3891-s001.docx]

**Liraglutide improves peripheral perfusion and markers of angiogenesis and inflammation in people with type 2 diabetes and peripheral artery disease: An 18-month follow-up of a randomised clinical trial**

**Supplementary material**

Figure 1…………………………………………………………………………………………………………………………………………………pag. 2

Table 1…………………………….……………………………………………………………………………………………………………………pag. 3


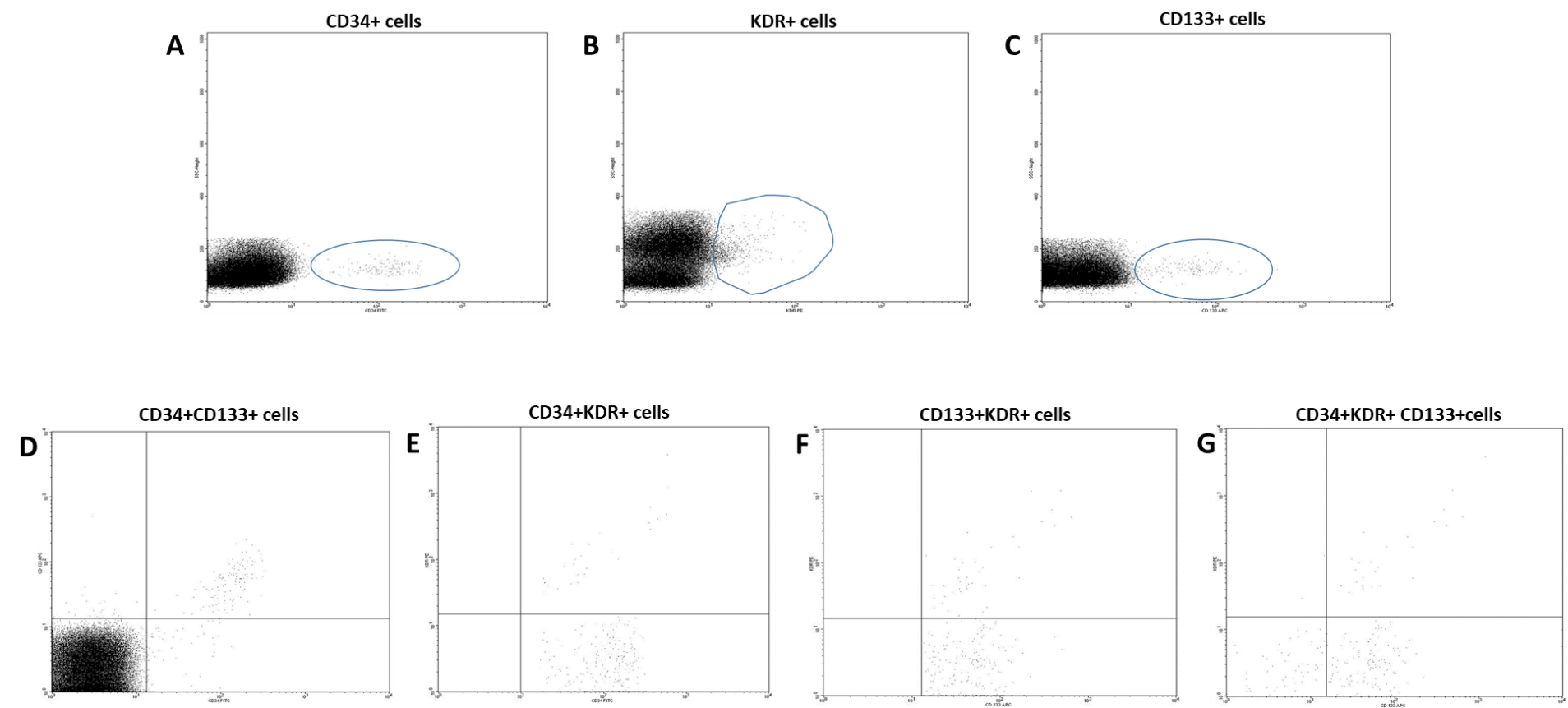


ESM Figure 1. A-G) Scatter plots illustrating the flow cytometry procedure used to quantify peripheral blood progenitor cells on the basis of the surface expression of CD34, CD133 and KDR.

ESM Table 1. Additional baseline characteristics.

|  | Liraglutide (27) | Control (28) |
| --- | --- | --- |
| Sex, n (%) |  |  |
| Male | 21 (78) | 22 (79) |
| Female | 6 (22) | 6 (21) |
| Tobacco, n (%) |  |  |
| Smokers | 12 (44) | 11 (39) |
| Not smokers | 4 (15) | 5 (18) |
| Ex-smokers | 11 (41) | 12 (43) |
| BMI, kg/m^2^ | 29.9 (4.0) | 28.1 (4.4) |
| Waist circumference, cm | 110.6 ± 8.6 | 106.1 ± 10.0 |
| Fasting Glucose, mg/dl | 132.0 (103.0, 152.5) | 123.0 (111.0, 137.0) |
| Fibrinogen, mg/dl | 432.0 (329.0, 495.5) | 406.0 (306.0, 471.0) |
| Renal Function |  |  |
| Creatinine, mg/dl | 0.9 (0.8, 1.4) | 0.9 (0.8, 1.2) |
| Azotemia, mg/dl | 46.5 (28.0, 71.0) | 38.0 (30.0, 50.0) |
| Anti-diabetes Therapy, n (%) |  |  |
| Metformin | 18 (75) | 20 (77) |
| Insulin | 13 (54) | 16 (62) |
| Other oral drugs | 7 (29) | 8 (31) |
| Hypertension Therapy, n (%) |  |  |
| ACE inhibitors or ARBs | 18 (75) | 18 (69) |
| β-blockers | 12 (50) | 12 (46) |
| α-blockers | 2 (8) | 2 (8) |
| Calcium channel blockers | 8 (33) | 12 (46) |
| Diuretics | 7 (29) | 9 (35) |
| Lipid-lowering Therapy, n (%) |  |  |
| Statins | 18 (75) | 20 (77) |
| Ezetimibe | 6 (25) | 6 (23) |
| Omega-3 | 4 (17) | 4 (15) |
| Fenofibrate | 2 (8) | 3 (12) |
| Antiplatelet Therapy, n (%) | 12 (50) | 16 (62) |
| Anticoagulant Therapy, n (%) | 12 (50) | 16 (62) |
| Previous PAD treatment, n (%) |  |  |
| PTA | 4 (15) | 3 (12) |
| Stenting | 2 (8) | 2 (8) |
| Bypass | 2 (8) | 3 (12) |
| PAD diagnostic procedure, n (%) |  |  |
| Doppler ultrasound | 10 (37) | 11 (39) |
| Angio-CT | 8 (30) | 7 (25) |
| Angiography | 9 (33) | 10 (36) |

Data are presented as mean ± SD, median (IQR) or number (percentage). ARB, angiotensin receptor blocker; BMI, body mass index; PAD, peripheral artery disease; PTA, percutaneous transluminal angiography.
